# Critical Care Needs in Patients with Diffusion-Weighted Imaging Negative MRI after tPA - Does One Size Fit All?

**DOI:** 10.1371/journal.pone.0141204

**Published:** 2015-10-30

**Authors:** Roland Faigle, Elisabeth B. Marsh, Rafael H. Llinas, Victor C. Urrutia

**Affiliations:** Department of Neurology, Johns Hopkins University School of Medicine, Baltimore, Maryland, United States of America; Osaka University Graduate School of Medicine, JAPAN

## Abstract

**Background and Purpose:**

Patients who receive intravenous (IV) tissue plasminogen activator (tPA) for ischemic stroke are currently monitored in an intensive care unit (ICU) or a comparable stroke unit for at least 24 hours due to the high frequency of neurological exams and vital sign checks. The present study evaluates ICU needs in patients with diffusion-weighted imaging (DWI) negative MRI after IV tPA.

**Methods:**

A retrospective chart review was performed for 209 patients who received IV tPA for acute stroke. Data on stroke risk factors, physiologic parameters, stroke severity, MRI characteristics, and final diagnosis were collected. The timing and nature of ICU interventions, if needed, was recorded. Multivariable logistic regression was used to determine factors associated with subsequent ICU needs.

**Results:**

Patients with cerebral infarct on MRI after tPA had over 9 times higher odds of requiring ICU care compared to patients with DWI negative MRI (OR 9.2, 95% CI 2.49–34.15). All DWI negative patients requiring ICU care did so by the end of tPA infusion (p = 0.006). Among patients with DWI negative MRI, need for ICU interventions was associated with higher NIH Stroke Scale (NIHSS) scores (p<0.001), uncontrolled hypertension (p<0.001), seizure at onset (p = 0.002), and reduced estimated glomerular filtration rate (eGFR) (p = 0.010).

**Conclusions:**

Only a small number of DWI negative patients required ICU care. In patients without critical care needs by the end of thrombolysis, post-tPA MRI may be considered for triaging DWI negative patients to a less resource intense monitoring environment.

## Introduction

Intravenous (IV) thrombolysis with recombinant tissue plasminogen activator (tPA) is a proven therapy for ischemic stroke in patients presenting within 4.5 hours of symptom onset [1]. Currently all post-tPA patients are monitored in an intensive care unit (ICU) or comparable stroke unit with ICU-like capabilities given their need for frequent vital sign checks and neurological examinations [2]. However, it is unclear whether routine ICU-admission or intensive monitoring is medically necessary for all post-tPA patients.

TPA is most effective when administered as early as possible, requiring rapid and expedited evaluation in the Emergency Department (ED). Other conditions, such as seizure, hypoglycemia, migraine, and conversion disorder may mimic symptoms of acute ischemic stroke. Additionally, patients presenting with cerebral ischemia may experience rapid resolution of their deficits with no evidence of infarct on post-tPA imaging. This is often referred to as neuroimaging-negative cerebral ischemia (NNCI), indicative of either a transient ischemic attack (TIA) or stroke averted by tPA [3]. Distinguishing stroke mimics from NNCI acutely is subjective and lacks diagnostic certainty; however, neither entity characteristically has evidence of cerebral infarction on post-tPA diffusion-weighted imaging (DWI). In order to expedite treatment, MR imaging is not typically employed prior to tPA administration for patients presenting with a presumed ischemic stroke. This results in tPA administration to patients with negative post-tPA DWI imaging at a rate of up to 26% [4–7]. Previous studies have shown that tPA administration in stroke mimics and NNCI is relatively safe, with rates of symptomatic ICH and mortality between 0% and 2% [5,7]. It remains unclear whether critical care needs in these patients differ from neuroimaging positive ischemic strokes, and whether ongoing ICU care for patients without evidence of cerebral infarction on post-tPA imaging is necessary.

In times of soaring health care costs, appropriate utilization of ICU resources is of vital importance. We have previously identified NIH Stroke Scale (NIHSS), African American race, and systolic blood pressure at presentation as independent predictors of ICU need after IV tPA [8], however, the role of neuroimaging in determining need for ICU care has not been addressed previously. The purpose of the present study was to assess the need for ICU care in patients receiving IV tPA for presumed acute ischemic stroke, who were subsequently found to have no evidence of infarction on post-tPA imaging.

To our knowledge, this is the first study to explore ICU needs in stroke mimics and NNCI post-IV thrombolysis.

## Methods

### Patients and study design

This study was approved by the Johns Hopkins University School of Medicine Institutional Review Board. Data was obtained from prospectively collected de-identified databases of patients treated for stroke at The Johns Hopkins Hospital and Johns Hopkins Bayview Medical Center. A waiver of consent was granted based on 45 CFR 46.116. An IRB waiver of HIPAA privacy authorization was also granted to allow review of medical records to abstract data to de-identify for use in research.

We retrospectively analyzed the medical records of all patients treated with IV tPA for presumed acute ischemic stroke in the ED at the Johns Hopkins Hospital and Johns Hopkins Bayview Medical Center between January 2010 and November 2013. Patients with in-hospital strokes and patients who were subsequently transferred to or from other hospitals after tPA administration were excluded. Demographic data including age, sex, and race were collected for all patients. Other variables of interest included stroke risk factors: hypertension, hyperlipidemia, diabetes mellitus, smoking status, history of atrial fibrillation, prior history of stroke, reduced ejection fraction (EF) (≤35%), and the pre-hospital use of antiplatelet agents, anticoagulation, and statins. NIHSS, presence or absence of seizure at onset, and the following physiologic parameters at presentation were recorded: blood pressure, blood glucose, and estimated glomerular filtration rate (eGFR) by Modification of Diet in Renal Disease (MDRD) equation [9]. The presence and time of any critical care intervention was recorded. A critical care intervention was considered any therapy or intervention that required ICU resources as defined previously [8]. Symptomatic intracerebral hemorrhage (sICH) was defined as any ICH with neurological deterioration, as indicated by a change in NIHSS ≥ 4 compared to the baseline as described previously [10].

All patients underwent neuroimaging prior to tPA administration as part of routine care. After tPA administration, all patients underwent follow-up MRI and/or CT. Patients were considered neuroimaging positive if either MRI or CT showed an infarct post-tPA. For patients with persistent neurological deficits despite no diffusion abnormality on initial negative MRI, repeat imaging was performed 24–48 hours later to confirm the presence of ischemic stroke, unless additional history and clinical information clearly suggested the presence of a stroke mimic [11,12]. Patients with neuroimaging consistent with cerebral infarct were compared to patients with DWI negative MRI. Given the relatively low sensitivity of head CT in the diagnosis of acute stroke, for the purposes of this study, patients who were unable to undergo MRI and who had no CT-evidence of stroke were excluded from analysis.

The diagnosis of a stroke mimic was based on absence of ischemia on post-tPA neuroimaging in addition to an alternative, more likely diagnosis found during work-up (eg. migraine). The diagnosis of NNCI was made by a vascular neurologist based on clinical presentation, examination, risk factor profile, and stroke work-up (eg. high grade carotid stenosis) consistent with cerebral ischemia, in the absence of a DWI positive lesion.

### Statistical Analysis

Statistical analysis was performed using Stata version 13 (*Stata Statistical Software*: *Release 13*. College Station, TX). A p-value of <0.05 was considered statistically significant. Continuous variables were analyzed using Student’s t-tests for normally distributed variables, and Wilcoxon rank-sum tests (Mann-Whitney U test) for non-normally distributed variables. Categorical variables were analyzed using Pearson’s Chi2 analysis, and Fisher’s exact tests, when appropriate.

Multivariable logistic regression was used to evaluate the relationship between DWI negative MRI and need for ICU care. The primary outcome of interest was need for any ICU intervention. Absence of stroke on MRI was the primary predictor of interest. Multivariable logistic regression was performed adjusting for basic demographic variables including age, sex, and race. Other variables felt to be clinically important for predicting critical care needs based on univariate analysis were subsequently included: stroke risk factors (hypertension, hyperlipidemia, diabetes mellitus, atrial fibrillation, smoking, reduced EF, and prior history of stroke or TIA), NIHSS, reduced eGFR (< 60 mL/min per 1.73 m^2^), and systolic blood pressure (SBP).

## Results

### Patient characteristics

A total of 209 patients received IV tPA in the ED between January 2010 and November 2013. One patient was excluded because he was transferred to another institution shortly after tPA administration. Another 6 patients were excluded because no post-tPA MRI data were available, leaving 202 patients for analysis.

The average age for all patients was 65.3 years (range 28–94); 51.5% were female; and 48.5% were African American. The mean NIHSS was 9.4. One hundred fifty-nine patients (78.7%) had a history of hypertension, 101 (50.0%) had a history of hyperlipidemia, 55 (27.2%) had a history of diabetes mellitus, 41 (20.3%) had a history of atrial fibrillation, and 29 (14.4%) had a history of reduced EF. The mean SBP at presentation was 163 mmHg, and the mean serum glucose upon presentation was 138 mg/dL. Further baseline patient characteristics are presented in Table 1.

A total of 54 patients (26.7%) were found to have no infarct on post-tPA MRI. Of those, about half (26; 48.2%) were diagnosed with NNCI. The most common etiologies for stroke mimics were migraine (10; 18.5%), conversion disorder (10; 18.5%), and seizure (4; 7.4%).

**Table 1:** Clinical characteristics, ICU needs, and outcomes of post-tPA patients with positive and negative neuroimaging. BP: blood pressure; SBP: systolic BP; DBP: diastolic BP; eGFR: estimated glomerular filtration rate; EF: ejection fraction; ICU: intensive care unit; LOS: length of stay; TIA: transient ischemic attack. P-values compare imaging positive and imaging negative patients by t-test/Wilcoxon rank-sum test for continuous variables, and Chi2/Fisher's exact test for categorical variables.

Characteristics	All patients *(n = 202)*	Imaging positive *(n = 148)*	Imaging negative *(n = 54)*	p-value
**Age**—years: mean (SD)	65.3 (15.5)	67.5 (15.2)	59.2 (15.1)	**<0.001**
range	28–94	28–94	29–89	
**Race**—n (%)				0.933
African American	98 (48.5)	71 (48.0)	27 (50.0)	
White	102 (50.5)	75 (50.6)	27 (50.0)	
Other	2 (1.0)	2 (1.4)	0 (0)	
**Sex**—female n (%)	104 (51.5)	67 (45.3)	37 (68.5)	**0.003**
**NIHSS**—mean (SD)	9.4 (5.8)	10.3 (6.1)	7.0 (4.2)	**<0.001**
**BP**—mm Hg: mean (SD)				
SBP	163 (32.9)	164 (31.1)	162 (37.7)	0.778
DBP	91 (19.5)	91 (19.7)	91 (19.1)	0.886
**tPA time window**—n (%)				
< 3 hours	150 (74.3)	114 (77.0)	36 (66.7)	0.136
**Glucose**—mg/dl: mean (SD)	138 (59.7)	141 (62.6)	130 (50.3)	0.249
**eGFR < 60 ml/min**—n (%)	67 (33.2)	54 (36.5)	13 (24.1)	0.097
**Risk factors for stroke**—n (%)				
Hypertension	159 (78.7)	117 (79.5)	42 (77.8)	0.845
Hyperlipidemia	101 (50.0)	77 (52.0)	24 (44.4)	0.340
Diabetes mellitus	55 (27.2)	40 (27.0)	15 (27.8)	0.916
Atrial fibrillation	41 (20.3)	37 (25.0)	4 (7.4)	**0.006**
Prior ischemic stroke/TIA	53 (26.2)	34 (23.0)	19 (35.2)	0.081
Current smoking	66 (32.7)	54 (36.5)	12 (22.2)	0.056
Reduced EF (≤35%)	29 (14.4)	25 (16.9)	4 (7.4)	0.089
**Medications**—n (%)				
Antiplatelet agent	89 (44.1)	66 (44.6)	23 (42.6)	0.800
Anticoagulation	15 (7.3)	14 (9.5)	1 (1.9)	0.075
Statin	80 (39.6)	57 (38.5)	23 (42.6)	0.600
**Any ICU intervention**—n (%)	59 (29.2)	54 (36.5)	5 (9.3)	**<0.001**
**Onset of intervention**—n (%)				
By end of tPA infusion	46 (22.8)	41 (27.7)	5 (9.3)	**0.006**
In first 24 hrs after tPA	10 (5.0)	10 (6.8)	0 (0)	**0.031**
Beyond 24 hrs post tPA	3 (1.5)	3 (2.0)	0 (0)	0.551
**Nature of intervention**—n (%)				
IV antihypertensives	23 (11.4)	19 (12.8)	4 (7.4)	0.282
Respiratory compromise	27 (13.4)	25 (16.9)	2 (3.7)	**0.015**
BP augmentation	5 (2.5)	5 (3.4)	0 (0)	0.327
Intraarterial therapy	10 (5.0)	10 (6.8)	0 (0)	0.065
Cerebral edema therapy	11 (5.5)	11 (7.4)	0 (0)	**0.039**
IV heart rate control	4 (2.0)	4 (2.7)	0 (0)	0.575
Angioedema	3 (1.5)	3 (2.0)	0 (0)	0.566
Intracerebral hemorrhage				
Symptomatic	4 (2.0)	4 (2.7)	0 (0)	0.575
Any	22 (10.9)	22 (14.9)	0 (0)	**0.003**
Other	3 (1.5)	3 (2.0	0 (0)	0.566
**Total LOS**—days: median (IQR)	5 (3–7)	5 (3.5–11)	3 (2–5)	**<0.001**
**Length of ICU stay**—n (%)				
> 2 days	37 (18.3)	35 (23.7)	2 (3.7)	**0.001**
**Discharge to**—n (%)				**<0.001**
Home	105 (55.9)	62 (46.3)	43 (79.6)	
Acute Rehab	51 (27.1)	45 (33.6)	6 (11.1)	
Subacute Rehab	32 (17.0)	27 (20.1)	5 (9.3)	
**Mortality**—n (%)	14 (6.9)	14 (9.5)	0 (0)	**0.019**

The median time to imaging after tPA was 12 hrs in the neuroimaging positive group, and 18 hrs in the neuroimaging negative group. Demographics of patients with negative DWI after tPA were compared to patients with subsequent imaging consistent with acute infarct (Table 1). Patients with negative DWI after tPA were more likely to be younger (mean age 59.2 ± 15.1 years vs. 67.5 ± 15.2 years; p<0.001), and female (68.5% vs. 45.3%; p = 0.003). The mean admission NIHSS in the group with positive neuroimaging after tPA was 10.3, and 7.0 in the neuroimaging negative group (p<0.001). The neuroimaging negative group had significantly lower rates of atrial fibrillation (7.4% vs. 25.0%; p = 0.006).

### ICU needs and outcomes in patients with DWI negative MRI after tPA

Of the 54 patients with DWI negative imaging after tPA, only 5 patients (9.3%) required ICU-level intervention. Fifty-four (36.5%) of the patients in the neuroimaging positive group required ICU care. All 5 patients in the neuroimaging negative group who required ICU interventions did so by the end of tPA infusion. Table 1 details the nature of ICU interventions in patients with and without infarct on subsequent imaging. The most common indications for ICU intervention in all patients were IV antihypertensives (11.4%) and respiratory compromise (13.4%). In the group with negative DWI, 4 patients (7.4%) required ICU intervention for uncontrolled hypertension, while 2 patients (3.7%) required respiratory support. Over the course of admission, the rate of sICH in the neuroimaging-positive group was 2.7%. The rate of any ICH was 14.9%; however, since our patients do not undergo routine imaging to evaluate for asymptomatic ICH prior to discharge, this number may underestimate the true rate of ICH. None of the patients in the DWI negative group experienced any ICH. Only 2 patients (3.7%) in the imaging negative group were in the ICU longer than 2 days, while 23.7% of patients with cerebral infarction on imaging had ICU stays exceeding 2 days. The median length of stay in the neuroimaging negative group after tPA was 3 days (interquartile range [IQR] 2–5), and was significantly lower than in the imaging positive group (5 days, IQR 3.5–11; p<0.001). Seventy-nine percent of patients without evidence of infarct on post-tPA imaging were discharged to home, while only 62 patients (46.3%) in the imaging positive group were able to go home at discharge (p<0.001).

In order to evaluate whether DWI negative MRI is an independent negative predictor of ICU needs post-tPA, we performed multivariable logistic regression. Patients with evidence of infarct on post-tPA imaging had over five times higher odds of requiring ICU interventions compared to patients with DWI negative MRI (odds ratio [OR] 5.6, 95% confidence interval [CI] 2.11–14.99) after adjusting for basic demographic variables, including age, sex, and race. The association was strengthened after adjusting for NIHSS, reduced eGFR, SBP, and other common stroke risk factors such as hypertension, diabetes, hyperlipidemia, atrial fibrillation, smoking, reduced EF, and prior stroke/TIA (OR 9.2, 95% CI 2.49–34.15).

### Characteristic of patients with DWI negative MRI after tPA requiring ICU resources

Among all patients with DWI negative MRI after tPA, we compared the characteristics of the five patients who required ICU interventions with the 49 patients who remained without need for ICU care (Table 2). The two groups were fairly similar; however, the mean NIHSS of patients requiring ICU care was 14.2, compared to 6.3 in patients with no ICU requirements (p<0.001). Patients requiring ICU interventions were more likely to present with seizure at onset (p = 0.002), uncontrolled hypertension (mean SBP 232 mmHg ± 40 vs. 155 mmHg ±30; p<0.001), or eGFR<60 mL/min per 1.73 m^2^ (p = 0.010). There was no significant difference for other demographic variables (age, race, gender, blood glucose), or frequency of common stroke risk factors (hypertension, hyperlipidemia, diabetes mellitus, atrial fibrillation, prior stroke or TIA, current smoking, or reduced EF) (Table 2).

**Table 2:** Demographic and clinical variables of patients with DWI negative MRI after tPA requiring critical care resources. ICU: intensive care unit; BP: blood pressure; SBP: systolic BP; DBP: diastolic BP; eGFR: estimated glomerular filtration rate; EF: ejection fraction. P-values compare patients with and without ICU needs by t-test for continuous variables, and Fisher's exact test for categorical variables.

Characteristics	No ICU need *(n = 49)*	ICU need *(n = 5)*	p-value
**Age**—years: mean (SD)	58.8 (15.0)	63.4 (17.5)	0.522
**Race**—white n (%)	26 (53.1)	1 (20.0)	0.351
**Gender**—female n (%)	34 (69.4)	3 (60.0)	0.645
**NIHSS**—mean (SD)	6.3 (5.4)	14.2 (7.9)	**<0.001**
**tPA time window**—n (%)			
< 3 hours	32 (65.3)	4 (80.0)	0.655
**Seizure at onset**—n (%)	1 (2.0)	3 (60.0)	**0.002**
**BP**—mm Hg: mean (SD)			
SBP	155 (29.7)	232 (39.6)	**<0.001**
DBP	88 (15.3)	124 (23.1)	**<0.001**
**Glucose**—mg/dl: mean (SD)	129 (52.2)	137 (26.7)	0.749
**eGFR < 60 ml/min/1.73m** ^**2**^—n (%)	9 (18.4)	4 (80.0)	**0.010**
**Risk factors for stroke**—n (%)			
Hypertension	37 (75.5)	5 (100)	0.575
Hyperlipidemia	21 (42.9)	3 (60.0)	0.646
Diabetes mellitus	13 (26.5)	2 (40.0)	0.610
Atrial fibrillation	4 (8.2)	0 (0)	1.000
Prior ischemic stroke/TIA	16 (32.7)	1 (20.0)	0.332
Current smoking	11 (22.5)	1 (20.0)	1.000
Reduced EF (≤35%)	4 (8.2)	0 (0)	1.000
**Medications**—n (%)			
Antiplatelet agent	22 (44.9)	1 (20.0)	0.380
Anticoagulation	1 (2.0)	0 (0)	1.000
Statin	20 (40.8)	3 (60.0)	0.640

## Discussion

Currently, all post-tPA patients are monitored in an ICU or stroke unit with ICU-like capabilities for at least 24 hours for intense post-tPA monitoring regardless of patient demographics, or other clinical or physiological variables. However, little data exist as to whether this “one size fits all” approach is medically necessary.

We have previously identified African American race, NIHSS, and systolic blood pressure as predictors of need for ICU care post tPA [8]. In the present study, we utilized neuroimaging information and show that only a small number of patients with negative post-tPA DWI-MRI require ICU interventions. Patients with DWI negative MRI after tPA represent the composite of NNCI and stroke mimics. For the purpose of investigating critical care needs post-tPA, we focused on dichotomizing patients based on DWI-MRI rather than “stroke vs. mimics” or “stroke vs. NNCI vs. mimics” for several reasons. DWI is objective and can be obtained relatively quickly where available, allowing for swift triaging decisions for post-tPA patients. In addition, differentiating between NNCI and mimics is often subjective in the acute setting, requiring collateral history, serial neurological examinations, additional testing, observation over time, and expert opinion. The incidence of stroke mimics and NNCI varies greatly among different studies [4–7]. This is in part due to lack of standardized definitions. In our study population, 12.9% were determined to have NNCI, and 13.9% stroke mimics. Thus, the combined incidence of stroke mimics and NNCI in our post-tPA population was about 27%, consistent with previous reports [4,7]. Interestingly, all five patients requiring critical care interventions in the MRI negative group were stroke mimics. Three patients presented with seizure, of which one required a drip for blood pressure control, one required intubation for airway protection, and one required both. The other two patients were diagnosed with conversion disorder by the primary team. Both required drips for blood pressure control. While this finding was somewhat unexpected, it may highlight the difficulty differentiating NNCI from stroke mimics. Patients with seizure at onset are particularly challenging since absence of DWI positivity after tPA might prompt some clinicians to diagnose a stroke mimic, while others would argue that a stroke with seizure at onset was successfully aborted with tPA before DWI changes have occurred.

Patients without post-tPA infarction on DWI were more likely to be young and female. This finding is consistent with previous reports [5,7], and may in part be related to a higher rate of migraine and conversion disorder in this population. In addition, patients with no evidence of infarction on post-tPA MRI presented with lower NIHSS, had shorter hospital stays, and were more likely to be discharged to home. Among patients with negative DWI after tPA, only 9.3% required an ICU intervention. Those patients required ICU needs for medical complications evident at presentation, including blood pressure control and respiratory compromise. Thus, our data demonstrate that DWI negative patients who do not require ICU care by the end of the tPA infusion do not require ICU care later on.

Within the DWI negative group, patients requiring ICU care were also more likely to have renal impairment on admission compared to patients without ICU needs. This was statistically significant despite the relatively small number of patients. This is consistent with previous reports suggesting that renal impairment in patients receiving IV tPA is independently associated with hemorrhagic transformation, poor outcome, and increased mortality [13–15].

Our study has several limitations. Post-tPA imaging was not obtained uniformly at pre-specified time points. The median tPA-to-imaging time in our population was 15 hours. False negative DWI studies have been described in as many as 6% of patients presenting with prolonged deficits greater than 24 hours [12]. However, none of our patients diagnosed with NNCI had symptoms beyond 24 hours, and in absence of a clear alternate diagnosis, the majority of patients with initially negative DWI-MRI imaging underwent repeat MRI at a later time point to confirm the absence of ischemia. Alternatively, while pre-tPA DWI was not routinely obtained in our patient population, it is unlikely that any delay in MRI resulted in falsely classifying an initial DWI positive MRI as DWI negative, since the rate of reversal of pre-tPA positive DWI has previously been reported to be less than 1% [16]. Thus, the likelihood of potentially misclassifying an imaging-positive ischemic stroke as mimic or NNCI is small. Our study population was derived from two single stroke centers over the course of just over 3.5 years. Therefore, extrapolating our results to community hospitals must be done with caution. While ICU interventions, procedures, and medication administration were well documented in the vast majority of cases, relying on accuracy of medical records has the potential to result in missing or inaccurate information by virtue of the retrospective nature of this study. In addition, MRI is not universally and readily available at all institutions, potentially limiting generalizability. Several institutions currently monitor post-PA patients in intermediate care units or dedicated stroke units. However, in order to comply with current guidelines, these patients are still required to undergo intense monitoring often with one-to-one nursing care. Independent of physical location of the patient, the high frequency of neurological exams and vital sign checks dictates resource-intense monitoring similar to ICU care.

The development of a patient profile that enables clinicians to identify patients without need for ICU resources after IV tPA would allow for optimization of resource utilization, potentially increasing cost effectiveness. Current guidelines would suggest that vital sign checks and neurological exams be continued in a standardized way for all post-tPA patients, and transfer to the regular floor would be feasible only after 24 hours post-tPA monitoring has been completed. In our DWI negative population, such intense monitoring at no point triggered an ICU intervention, and did not change the course of clinical care. All DWI negative patients requiring ICU interventions did so for blood pressure control and/or intubation for airway protection, and all were identified either during or prior to tPA administration. Therefore it may be reasonable to use the lack of DWI abnormality on MRI as a triage tool to determine the level of post-tPA monitoring intensity. Further prospective studies are needed to establish that less intense monitoring of select subgroups of post-tPA patients is reasonable and safe.
